# Transient receptor potential vanilloid 1: A potential therapeutic target for the treatment of osteoarthritis and rheumatoid arthritis

**DOI:** 10.1111/cpr.13569

**Published:** 2023-11-23

**Authors:** Zhidong Liao, Muhammad Umar, Xingyun Huang, Ling Qin, Guozhi Xiao, Yan Chen, Liping Tong, Di Chen

**Affiliations:** ^1^ Department of Bone and Joint Surgery the First Affiliated Hospital of Guangxi Medical University Nanning China; ^2^ Research Center for Computer‐aided Drug Discovery, Shenzhen Institute of Advanced Technology Chinese Academy of Sciences Shenzhen China; ^3^ Faculty of Pharmaceutical Sciences Shenzhen Institute of Advanced Technology Shenzhen China; ^4^ Collaborative Innovation Centre of Regenerative Medicine and Medical BioResource Development and Application Co‐constructed by the Province and Ministry Guangxi Medical University Nanning Guangxi China; ^5^ Musculoskeletal Research Laboratory of Department of Orthopaedics & Traumatology and Innovative Orthopaedic Biomaterial & Drug Translational Research Laboratory Li Ka Shing Institute of Health Sciences, The Chinese University of Hong Kong Hong Kong China; ^6^ School of Medicine Southern University of Science and Technology Shenzhen China

## Abstract

This study aims to determine the molecular mechanisms and analgesic effects of transient receptor potential vanilloid 1 (TRPV1) in the treatments of osteoarthritis (OA) and rheumatoid arthritis (RA). We summarize and analyse current studies regarding the biological functions and mechanisms of TRPV1 in arthritis. We search and analyse the related literature in Google Scholar, Web of Science and PubMed databases from inception to September 2023 through the multi‐combination of keywords like ‘TRPV1’, ‘ion channel’, ‘osteoarthritis’, ‘rheumatoid arthritis’ and ‘pain’. TRPV1 plays a crucial role in regulating downstream gene expression and maintaining cellular function and homeostasis, especially in chondrocytes, synovial fibroblasts, macrophages and osteoclasts. In addition, TRPV1 is located in sensory nerve endings and plays an important role in nerve sensitization, defunctionalization or central sensitization. TRPV1 is a non‐selective cation channel protein. Extensive evidence in recent years has established the significant involvement of TRPV1 in the development of arthritis pain and inflammation, positioning it as a promising therapeutic target for arthritis. TRPV1 likely represents a feasible therapeutic target for the treatment of OA and RA.

## Graphical abstract

TRPV1, a member of the TRP family, is widely expressed in primary sensory neurons and various cells and participates in pain signal transduction of thermal, mechanical and chemical stimulation. Recently, functional changes in TRPV1 channels have been considered as risk factors for OA and RA. Abnormal expression and function of TRPV1 channels are associated with chondrocyte ferroptosis, synovial inflammatory response, macrophage polarization and bone homeostasis. In addition, TRPV1 channels play important roles in neural sensitization, defunctionalization or central sensitization and are involved in the occurrence of pain in OA and RA. Based on clinical and preclinical evidence, TRPV1‐targeting drugs and regulation of its expression exhibit prominent curative effects in OA and RA. TRPV1 may be a novel potential therapeutic target for OA and RA treatment.

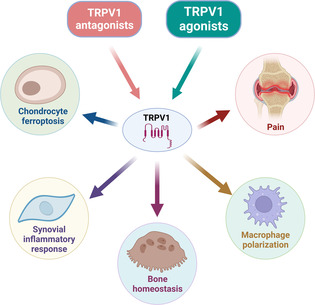

## 1 INTRODUCTION

Arthritis is a chronic disease that refers to the joint's inflammation involving progressive degenerative or inflammatory pathological changes in articular cartilage, synovium, subchondral bone and other tissues that eventually lead to joint disability. Arthritis is categorized into two major forms known as osteoarthritis (OA) and rheumatoid arthritis (RA) which remain the core reasons for disability and pain in the elderly. The stiffness and pain caused by arthritis limit an individual's joint function and affect their quality of life. Symptoms worsen gradually with age and deteriorate the joints as the immune system attacks the joints.^1^ In addition to pain, individuals with arthritis also experience other clinical symptoms, such as joint stiffness and dysfunction.^2^ The currently available treatment for arthritis mainly includes symptomatic treatment including non‐pharmacological rehabilitation (e.g., exercise and diet), pharmacological therapy and surgical intervention.^3^, ^4^ Drug therapy is generally used to reduce the symptoms of arthritis. Such drugs include non‐steroidal anti‐inflammatory drugs (NSAIDs), opioids, corticosteroids and even immunosuppressive agents; however, these drugs are found to be associated with adverse side effects.^5^, ^6^, ^7^ While TNF inhibitors, anti‐IL‐17/23 antibodies and Janus Kinase inhibitors have shown their efficacy in the management of RA, some of them are still associated with adverse side effects, including headache, hypertension, injection site reactions and allergies and a higher rate of upper respiratory tract infections.^8^, ^9^, ^10^ In addition, the side effects of drugs can even lead to the complete deterioration of joints.^11^ Therefore, there is an urgent need to identify the potential target molecules and develop new drugs for local or systemic applications for the treatment of arthritis.

Transient receptor potential (TRP) channels act as ‘molecular receptors’ of cells, composed of six subunits containing transmembrane domains, which were discovered in Drosophila.^12^ TRP channels respond to a variety of stimuli, including light, temperature, touch, osmotic pressure, taste, sound and pain.^13^ The TRP channel family is divided into six subfamilies and harbours 28 members in mammals, including TRP canonical (TRPC), TRP vanilloid (TRPV), TRP melastatin (TRPM), TRP polycystin (TRPP), TRP mucolipin (TRPML) and TRP ankyrin (TRPA) subfamilies.^14^ TRPV1, a member of the TRPV subfamily, is a non‐selective cation channel protein that can be activated by capsaicin, high temperature (>43°C), low PH (<6) and other harmful stimuli.^15^ After activation of TRPV1, calcium and sodium ions are allowed to flow into the cells, eventually leading to the release of molecules involved in pain transmission, such as calcitonin gene‐related peptide (CGRP), substance P,^16^ somatostatin,^17^ glutamate^18^ and bradykinin.^19^ TRP channel activation exacerbates local inflammation by releasing pro‐inflammatory mediators while also exerting anti‐inflammatory effects. Somatostatin employs systemic anti‐inflammatory and analgesic effects in Complete Freund's adjuvant (CFA)‐induced arthritis models^17^ and reduces matrix metallopeptidase (MMP) 1 production by synoviocytes from RA patients.^20^ It has been reported that TRPV1 is expressed in various cells, such as chondrocytes, synovial fibroblasts, osteoclasts and osteoblasts.^21^, ^22^, ^23^, ^24^, ^25^, ^26^, ^27^ TRPV1 is known to play an analgesic role in various diseases, including OA,^28^ RA,^29^ postherpetic neuralgia,^30^ diabetic neuropathy^31^ and cancer.^32^ TRPV1 not only serves as a source of anti‐ferroptosis in chondrocytes but also is imperative for inflammation and pain progression in arthritis.^33^, ^34^ Therefore, based on such characteristics and involvement in arthritis pain, TRPV1 is considered a potential target for the treatment of arthritis.

Here, we reviewed the current treatment status of OA and RA. We have discussed the expression and functional correlation of TRPV1 in different tissues under arthritis condition, as well as the role of TRPV1 in arthritis pain. Additionally, we briefly summarized the clinical application of TRPV1 agonists and antagonists in arthritis diseases.

## 2 CURRENT TREATMENT STATUS OF OA AND RA

Osteoarthritis (OA), the most common form of arthritis, is a chronic disease characterized by synovial inflammation, cartilage degradation, changes in subchondral bone mass and osteophyte formation.^35^, ^36^, ^37^ OA mainly affects the knee, hip, hands and spinal joints.^35^, ^38^, ^39^ The risk factors for OA include genetics, age, sex, congenital anatomical abnormalities and metabolic disorders.^40^ In addition, joint injury and mechanical damage are also responsible for the occurrence of OA.^41^ The number of OA patients is increasing yearly, especially with the aging population is significantly expanding in recent years, representing a substantial burden on patients' social and economic well‐being.^42^, ^43^ However, there are currently no effective treatment strategies available for OA. The purpose of all existing treatments is to relieve symptoms and prevent the complications and progression of the disease to improve the quality of life. Pain is one of the main symptoms of OA and can be relieved by taking NSAIDs and opioid drugs; nevertheless, their efficacy decreases over time. The long‐term use of oral NSAIDs increases the risk of gastrointestinal and cardiovascular side effects.^6^ In clinical practice, it is crucial to enhance personalized medication and monitoring for high‐risk and elderly individuals. Furthermore, opioids present significant challenges in terms of tolerance and safety risks, such as abuse potential and rapid tolerance.^7^ The use of opioids is linked to the progression of structural degeneration in knee joint.^44^ When individuals do not respond to oral or topical analgesics, intra‐articular corticosteroid injections are often administered,^45^ providing short‐term pain relief and improved function. However, prolonged usage of this drug fails to alleviate symptoms and may even worsen cartilage degeneration.^46^, ^47^ Total joint replacement is a viable treatment option for individuals with severe functional impairment, but it has certain limitations due to the longevity of the prosthesis and potential inflammatory reactions.^48^, ^49^ Total knee arthroplasty is not ideal for individuals with higher preoperative knee pain scores at rest owing to their lower pain threshold, increasing the risk of persistent pain after surgery.^50^ Considering these unsatisfactory outcomes, there is an urgent need to discover novel pharmacological approaches for the treatment of OA.

Rheumatoid Arthritis (RA) is an inflammatory autoimmune disease characterized by chronic inflammation of the synovium, leading to the destruction of cartilage and bone tissue in the joints. This progressive condition manifests through symptoms like joint pain, swelling and stiffness.^51^ Inflammatory cytokines produced by T cells, B cells, macrophages and synovial fibroblasts in RA, such as tumour necrosis factor‐alpha (TNF‐α), interleukin (IL)‐1 and IL‐17 aggravate the inflammatory response of the synovium and bone destruction.^52^ Early and active treatment with disease‐modifying antirheumatic drugs (DMARDs) has proven effective in slowing down the progression of RA and alleviating clinical symptoms.^53^ Currently, the main drugs used for the treatment of RA are NSAIDs, glucocorticoids, DMARDs and surgical therapy. DMARDs are divided into conventional synthetic (cs) DMARDs, biological (b) DMARDs and targeted synthetic (ts) DMARDs. Among them, methotrexate (MTX) is a widely used csDMARD and is considered a promising drug for RA treatment.^54^ In individuals with an inadequate methotrexate response, bDMARDs combined with methotrexate have shown superiority over bDMARDs monotherapy.^55^, ^56^ However, considering the potential long‐term immunosuppressive effects and increased risk of infection associated with MTX use, bDMARDs or tsDMARDs should be considered as alternative options for individuals with contraindications to MTX.^56^ Glucocorticoids remain common and essential treatment for RA, although their prolonged low‐dose usage raises the risk of cardiovascular disease, osteoporosis and fracture.^57^, ^58^, ^59^ With the rapid advancement of RA treatment drugs, the multifaceted selection and combination of drugs provide more treatment possibilities for the personalized treatment of individuals with RA. In cases of severe RA with joint dysfunction, deformity or insufficient relief from conservative treatment, surgical interventions are often considered the primary approach to alleviate disease progression.^60^

In the context of OA and RA, drug therapy plays a crucial role in managing symptoms and slowing disease progression. However, the effectiveness of many drugs for OA or RA treatment is limited due to individual resistance and contraindications, making the development of novel drugs with improved efficacy and fewer side effects a pressing need. In this regard, the TRPV1 channels, which belong to the ion channel family called TRP channels, act as a promising therapeutic target for the development of new drugs with enhanced efficacy and safety profiles.

## 3 ROLE OF TRPV1 IN ARTICULAR CARTILAGE

Chondrocytes are the sole cell type in articular cartilage, playing a crucial role in maintaining the normal physiological function of articular cartilage.^61^ In the pathophysiology of OA, inflammatory molecules like IL‐1β, TNF and IL‐6 act as critical mediators. Specifically, IL‐1β and TNF hinder chondrocyte anabolism and promote the release of proteolytic enzymes, thereby accelerating cartilage degeneration.^62^, ^63^ Valdes et al. demonstrated that TRPV1 gene expression in chondrocytes was increased in response to pro‐inflammatory cytokines IL‐1 and TNF‐α, suggesting that changes in TRPV1 expression in chondrocytes appear to play an important role in the structural damage in OA.^64^ However, only a few studies have evaluated the role of TRPV1 in cartilage function. Furthermore, various types of chondrocyte death, including cell necrosis, apoptosis, autophage^61^ and ferroptosis^65^ are involved in OA pathogenesis. Many previous studies have demonstrated that inhibition of TRPV1 channels can reduce chondrocyte degeneration, but the role of TRPV1 channel activity in arthritic chondrocytes is controversial. Interestingly, recent research in mice has indicated that the TRPV1 agonist capsaicin activates TRPV1 channels in OA chondrocytes, promoting the expression of the antioxidant enzyme glutathione peroxidase 4 (GPX4). GPX4 protects chondrocytes from ferroptosis and reduces cartilage degradation.^34^, ^66^, ^67^ GPX4 is a crucial regulator of ferroptosis, and the reduction in its expression leads to increased sensitivity of chondrocytes to oxidative stress and extracellular matrix degradation. These findings suggest a complex role for TRPV1 in maintaining cartilage homeostasis, necessitating further investigation into its mechanism and impact on chondrocyte function in OA.

Chondrocytes are known to respond to mechanical stimuli such as compression forces and shear stress. Excessive mechanical loading contributes to OA pathogenesis, leading to catabolic reactions and chondrocyte death.^68^ In vitro stimulation of chondrocytes with pro‐inflammatory cytokines has been utilized to mimic changes in cartilage degradation associated with OA.^62^, ^69^, ^70^ Inflammatory cytokines induce extracellular calcium ion (Ca^2+^) influx and nuclear translocation of nuclear factor‐kappa B (NF‐κB) in chondrocytes, exacerbating cartilage degradation.^71^ Mechanical strain promotes the efflux of intracellular Ca^2+^ through the TRPV1 channel, and the intracellular Ca^2+^ concentration decreases to inhibit NF‐κB activity, leading to a decrease in the expression of a disintegrin and metalloproteinase with thrombospondin motifs 9 (ADAMTS9) induced by inflammatory cytokines. Inhibition of TRPV1 activity using a mechanically gated channel inhibitor, such as capsazepine, abolishes the regulation of ADAMTS9 by mechanical strain, while inhibition of TRPV2 and TRPV4 has no such effect.^71^ Therefore, mechanical strain‐induced attenuation of ADAMTS9 expression in chondrocytes may be mainly achieved by TRPV1‐associated channels. It is commonly known the fact that cell migration is influenced by changes in intracellular Ca^2+^ concentration. TRPV1 is a non‐selective Ca^2+^ channel implicated in cell migration.^72^ Studies on high‐density micromass cell cultures in chickens and mice have demonstrated that mesenchymal cells expressing TRPV1 migrate and proliferate, contributing to cartilage formation, which implies that TRPV1 activity may affect chondrocyte proliferation and extracellular matrix production.^73^

## 4 ROLE OF TRPV1 IN SYNOVIUM

Cartilage degeneration and the production of cartilage fragments in arthritis promote the inflammatory response of the synovium and are often accompanied by infiltration of macrophages and T lymphocytes.^74^ These cells secrete cytokines and chemokines, exacerbating cartilage catabolism. Inflammatory factors can also promote angiogenesis.^75^ Simultaneously, a reduction of synovial fluid leads to increased friction between articular cartilages, forming a vicious circle that eventually leads to progressive joint degeneration.^76^, ^77^ Clinically, individuals with OA have a 50% chance to develop synovitis, which causes joint pain, hyperthermia and swelling.^78^ The severity of synovitis is strongly correlated with the progression of cartilage destruction in OA.^79^ Synovial fibroblasts are important cells of the joint synovium and play a crucial role in the inflammatory response of arthritis.^80^ In chronic arthritis induced by Complete Freund's Adjuvant, mice with TRPV1 receptor deletion showed significantly reduced joint swelling and cartilage destruction.^81^, ^82^ The expression of *TRPV1* mRNA and protein in synovial tissue is increased in human and rat arthritis models.^22^, ^23^, ^24^ TRPV1 activation in synovial fibroblasts can promote the expression of pro‐inflammatory cytokines and participate in the partial production of reactive oxygen species (ROS).^22^, ^83^, ^84^, ^85^ Conversely, the expression of TRPV1 can also be upregulated by pro‐inflammatory cytokines and ROS.^83^ It has been shown that subcutaneous injection of the peptide APHC3 (a TRPV1 antagonist) extracted from sea anemones reduced joint swelling, thermal and mechanical hypersensitivity of pain and improved grip strength in complete Freund's Adjuvant (CFA‐) and monosodium iodoacetate (MIA)‐induced OA.^86^ The destruction of articular cartilage was significantly reduced from day 8 to day 15, and the concentration of IL‐1β in synovial fluid was significantly reduced at day 15.^86^ The TRPV1 antagonist can reduce chondrocyte degeneration by inhibiting the joint inflammatory response, suggesting that targeting the TRPV1 channel is a potentially valid approach for the treatment of joint synovial inflammation.

Synovial macrophages are necessary for maintaining homeostasis within the joint. The abnormal aggregation of synovial macrophages in joints has been implicated in the pathogenesis of OA and RA,^87^ and the inhibition of macrophage generation and aggregation can reduce structural damage. Recent studies showed that the expression of macrophages and TRPV1 was increased in synovial tissues of the knee joint of individuals with RA and OA, and most TRPV1 is highly localized to CD68‐positive macrophages.^23^ Subsequently, another study showed that TRPV1 is always expressed in M1 macrophages,^24^ suggesting that TRPV1 may be related to the function of M1 macrophages. Intra‐articular injection of a TRPV1 agonist capsaicin activates TRPV1 and inhibits M1 macrophage polarization because the activation of TRPV1 induces large amounts of Ca^2+^ influx through TRPV1 channels. This increase in Ca^2+^ influx leads to the phosphorylation of intracellular calmodulin‐dependent protein kinase II (CaMKII), promotes the nuclear translocation of nuclear factor erythroid‐2 related factor 2 (Nrf2) and ultimately results in the reduction of macrophages polarization.^24^ Compared with the control group, the infiltration of M1 macrophages in the synovium of OA mice after capsaicin injection was reduced, the cartilage surface was smoother, and there was a reduction observed in both the surface fibrosis area and the loss of cartilage extracellular matrix.^24^ These findings suggest that, through activating the Ca^2+^/CaMKII/Nrf2 pathway, TRPV1 agonist has the ability to reduce M1 macrophage infiltration in the synovial tissue inflammation.

In addition to the identification of TRPV1 downstream signalling molecules, the upstream regulatory mechanisms of TRPV1 have also been reported by a recent study. It has been reported that TRPV1 is regulated by hyaluronic acid (HA).^88^ HA is continuously secreted by synovial lining cells into the joint fluid and is widely distributed in the extracellular matrix in mammals.^89^ HA affects the viscoelasticity of synovial fluid and plays an important role in the lubrication and cushioning of joints.^90^ Compared with normal joint synovial fluid, HA in synovial fluid usually depolymerizes from high to low molecular weight in OA, resulting in a decrease in the viscoelasticity of synovial fluid,^91^ which eventually leads to joint damage. Interestingly, Caires and colleagues found that administration of HA inhibited the opening of TRPV1 channels in nociceptive terminals, reduced the response to noxious stimuli and reduced joint pain.^88^ A reasonable explanation is that HA may normally maintain the TRPV1 channel of nociceptive terminals in a closed state. When arthritis occurs, HA elastoviscous filtering capacity decreases, reducing the regulation of the TRPV1 channel and increasing the excitability of peripheral nociceptive neurons.

## 5 ROLE OF TRPV1 IN SUBCHONDRAL BONE

Normal bone homeostasis depends not only on extracellular Ca^2+^ concentration but also on the regulation of multiple bone cells and their intracellular Ca^2+^ signalling.^92^ The dynamic balance between osteoblast‐mediated bone formation and osteoclast‐mediated bone resorption ensures the relative stability of the mechanical strength of trabecular bone, allowing joints to withstand sufficient stress.^93^ In OA, decreased osteoprotegerin secretion by osteocytes leads to enhanced osteoclast‐mediated bone resorption.^94^ With the increase in mechanical loading, the decrease in sclerostin secreted by osteocytes leads to the enhancement of the wingless‐type MMTV integration site (Wnt) signalling pathway, ultimately resulting in the enhancement of osteoblast‐mediated bone formation.^39^ In the late stage of OA, abnormal bone remodelling caused by joint biomechanical disturbances can lead to insufficient mineralization of subchondral bone, making it more vulnerable to destruction.^95^ The receptor activator of nuclear factor kappa‐B ligand (RANKL) plays a crucial role in bone loss in OA and RA. Osteoclasts can participate in the regulation of bone resorption through the RANKL‐RANK signalling pathway.^96^, ^97^, ^98^ Several studies have shown that TRPV1 is expressed in osteoblasts and osteoclasts and is involved in balancing bone resorption and formation.^26^, ^27^, ^99^ TRPV1 antagonists inhibited the osteoclast differentiation in vitro and reduced bone resorption and protected ovariectomy‐induced bone loss in mice.^27^ Increased bone volume fraction, trabecular thickness and bone mineral density were observed in TRPV1 knockout (KO) mice.^100^ This may be due to the fact that absence of TRPV1 inhibits extracellular Ca^2+^ influx into osteoclasts, leading to the down‐regulation of nuclear factor of activated T cells c1 (NFATc1), a downstream transcription factor for Ca^2+^ signalling and ultimately leads to the reduction of osteoclast formation.^100^ NFATc1 is a key downstream target of the RANKL‐RANK signalling pathway involved in osteoclastogenesis and plays a crucial role in regulating bone homeostasis.^101^ Therefore, TRPV1 channels participate in the regulation of bone remodelling by affecting NFATc1 activity during OA pathology. Pretreatment of rats with TRPV1 agonists, such as resiniferatoxin (RTX) causes desensitization of nerve endings expressing TRPV1 receptors without affecting the non‐neural TRPV1 channels.^102^ Pre‐treatment with capsaicin for 2 weeks could effectively inhibit the reduction of bone volume and trabecular number in MIA‐induced OA,^103^ indicating that capsaicin pre‐treatment reduces bone damage in early OA, such as the destruction of trabecular bone, but whether it is effective for end‐stage OA remains unclear. Furthermore, RTX pre‐treatment resulted in increased bone mass and thickness of the trabecular while reducing average trabecular separation in the collagen‐antibody‐induced RA mouse model, suggesting that defunctionalization of capsaicin‐sensitive afferents leads to decreased bone resorption during joint inflammation.^104^

Tissue acidosis is an essential pathological feature of arthritis, where inflammation of synovial tissue during arthritis progression leads to localized acidification, which may lead to changes in ion channel activity. With the increase in hydrogen ion concentration in RA synovial fluid, extracellular acidification can induce chondrocyte pyroptosis by activating acid‐sensing ion channel 1a (ASIC1a), leading to irreversible cartilage degradation.^105^, ^106^ Moderate extracellular acidification (pH >6) does not directly activate TRPV1 channels^107^ but it inhibits induced cell death by modulating Ca^2+^ and NF‐κB translocation and ROS production in synovial cells.^108^ Particularly, local acidosis promotes osteoclastogenesis and SB‐366791 (TRPV1 antagonist) inhibits osteoclast activation.^109^ Studies have also shown that bone loss can be reduced by simultaneously stimulating cannabinoid receptor 2 and blocking TRPV1 channels.^110^ These findings reveal TRPV1 as a potential therapeutic target for preventing bone loss and regulating bone mass in arthritis. Future studies may be aiming at investigation how TRPV1 expression in osteoclasts can be exploited to restore the balance between osteoclast and osteoblast bone homeostasis under pathological conditions of arthritis.

## 6 ROLE OF TRPV1 IN ARTHRITIS PAIN

TRPV1 is a critical ion channel involved in pain perception and is considered a potential target for the treatment of OA pain. The Ile585Val genetic variant in the coding region of the TRPV1 gene is associated with pain in individuals with symptomatic OA.^64^ Early studies suggested that compared with wild‐type counterparts, TRPV1 receptor KO (*Trpv1* ^*−/−*^) mice showed little response to inflammatory heat pain but a normal response to noisier mechanical stimuli.^111^, ^112^ Some recent studies have also reported that TRPV1 receptor gene‐deleted (*Trpv1* ^*−/−*^) mice reduce mechanical hyperalgesia.^81^, ^82^, ^113^ Similarly, TRPV1 antagonists were effective in suppressing arthritic mechanical allodynia.^114^ These studies suggest that the TRPV1 receptor is essential for maintaining mechanical hyperalgesia and joint inflammatory pain.

**FIGURE 1:**
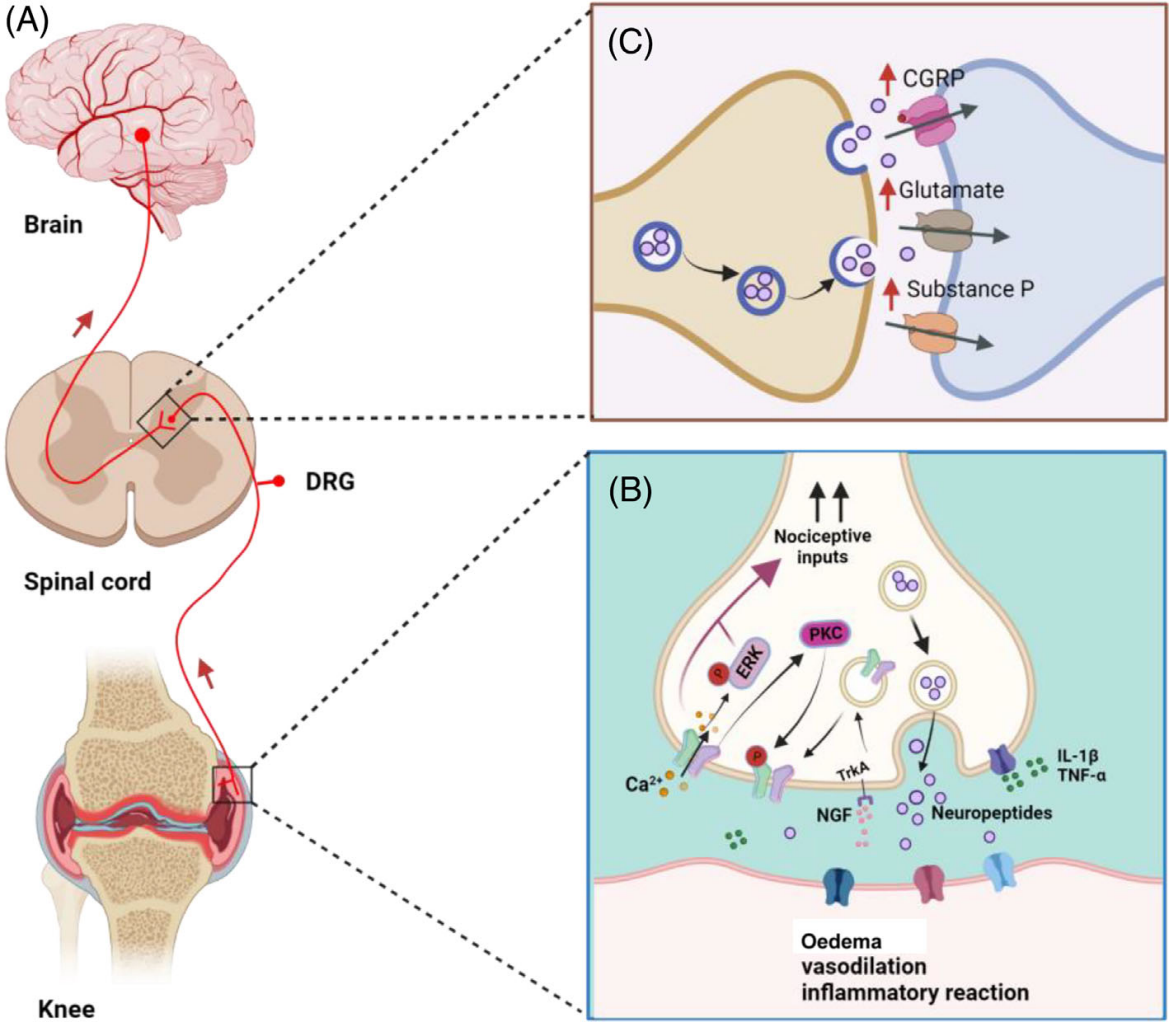
TRPV1 channels play an essential role in generating OA pain. (A) Nociceptive signals from nociceptors are transmitted to the spinal cord via unmyelinated C or myelinated A fibres. Nociceptive signals are transmitted from the dorsal horn of the spinal cord to higher centres and then to the cerebral cortex to produce pain sensation. (B) In response to inflammatory factors such as nerve growth factor (NGF), tumour necrosis factor‐alpha (TNF‐α) and interleukin (IL)‐1β, TRPV1 channels are activated to transmit pain signals. Protein kinase C (PKC) promotes TRPV1 phosphorylation, increasing sensitivity. Activation of TRPV1 channels leads to increased neurotransmitter release and extracellular regulated protein kinases (ERK) phosphorylation, leading to peripheral nerve sensitization. In addition, joint oedema, vasodilatation and inflammatory response occur under the action of inflammatory factors. (C) Nociceptive signals are transmitted along the dorsal root ganglion (DRG) and central synapses in the spinal cord release various neuropeptides (e.g., calcitonin‐gene‐related peptide (CGRP), glutamate or substance P) resulting in central sensitization.

The sensitization of peripheral afferents and central sensitization are key factors in the development of arthritis pain. According to different neurochemical characteristics, primary afferent nociceptors can be divided into peptidergic and non‐peptidergic neurons, and most TRPV1‐positive afferent nerves are peptidergic.^115^ TRPV1 is mainly located in C‐fibre and some Aδ‐fibre primary afferents,^116^ involved in the conduction of neurogenic and mechanical pain that innervates the knee and ankle.^117^ TRPV1 is highly enriched in nociceptors containing neuropeptides, such as afferents containing CGRP and substance P.^115^, ^118^ The co‐localization of TRPV1 and CGRP in afferent nerves is increased in OA and is thought to play a role in the perception of joint pain.^119^ CGRP is related to the pain of peripheral and central sensitization of arthritis and can promote the production of TNF‐α, IL‐1β and IL‐6 in monocytes, thereby increasing the inflammatory response of tissues and cartilage degradation.^120^, ^121^, ^122^, ^123^ In arthritis, nociceptor nerve endings can promote the release of neuropeptides (CGRP, substance P, glutamate and somatostatin) from C‐fibre neurons into the periphery by activating TRPV1.^17^, ^124^, ^125^ Subsequent nociceptor depolarization generates action potentials along the L3‐L5 dorsal root ganglion (DRG), causing the release of neurotransmitters in layers I and II of the dorsal horn of the spinal cord,^126^, ^127^ leading to central sensitization^128^ (Figure 1). Somatostatin exerts both anti‐inflammatory and analgesic effects in arthritis,^17^ suggesting that the analgesic effect induced by TRPV1 agonists may be partially attributed to somatostatin released from primary afferent nerves. Furthermore, local activation of metabotropic glutamate receptor 5 (mGluR5) in the infralimbic cortex (IL) is associated with thermal hyperalgesia in rodents.^129^ Spinal TRPV1 also participates in the descending pronociceptive effect induced by 5mGluR5 activation in mono‐arthritis (ARTH).^130^

The extracellular signal‐regulated kinase (ERK) is a widely expressed cell signalling molecule. ERK is phosphorylated in sensory neurons, thought to underlie the mechanism of thermal hyperalgesia.^131^ Studies have shown that arthritis pain is associated with the activation of the ERK pathway, and inhibition of this pathway reduces nociceptive behaviour and inflammatory response.^132^ Activation of TRPV1 channels can promote the release of CGRP and glutamate, thus increasing ERK phosphorylation in neurons, suggesting that TRPV1 may mediate the activation of ERK in the traumatic primary afferent nerves.^133^ During arthritis pain, multiple inflammatory mediators directly act on auto‐receptors and can enhance TRPV1 channel protein phosphorylation by activating various protein kinases. These mediators function as endogenous ligands of TRPV1 channels.^134^, ^135^, ^136^ Protein kinase Cε (PKCε) mediates the phosphorylation and dephosphorylation of TRPV1 at Ser502 and Ser800, playing an indispensable role in inflammation and neuropathic pain progression.^137^ In MIA‐induced arthritis rats, PKCɛ activates TRPV1 by inducing TRPV1 phosphorylation at Ser800, resulting in inflammatory hyperalgesia in the rats. However, TRPV1 at Ser502 is not affected, possibly due to the different functions of PKA and PKC in regulating TRPV1 in various neurons or diseases.^138^ Increased expression of inflammatory factors, such as nerve growth factor (NGF) in OA promotes TRPV1 phosphorylation and functional enhancement, leading to obvious hyperalgesia.^139^ Activation of the NGF receptor tyrosine kinase A (TrkA) at Y760 triggers the activation of Src kinase by phosphoinositide 3‐kinase, resulting in increased TRPV1 phosphorylation. This facilitates the translocation and insertion of TRPV1 into the cell membrane surface and ultimately induces hyperalgesia.^140^ In addition, several endogenous ligands, such as cannabinoids, linoleic acid metabolites and lysophosphatidic acid directly activate the TRPV1 channel to promote the release of neuropeptides from nerve endings. This results in inflammatory responses and the production of pain.^141^ This therapeutic approach may preserve drug efficacy while avoiding drug‐induced hyperthermia by mediating endogenous ligands, thereby inhibiting TRPV1 channel activity rather than directly inhibiting TRPV1 activity.

TRPV1 antagonists and activators serve as agents that contribute to the analgesic effects in arthritis (Table 1). TRPV1 antagonists such as A‐995662, A‐889425, JNJ‐17203212 or APHC3 reduce the excitability of peripheral afferent fibres (C and Aδ fibres) and inhibit the release of neuropeptides in the spinal cord, effectively reducing changes in pain‐related behaviour. These changes include thermal and mechanical hyperalgesia, asymmetric weight‐bearing of extremities and decreased hindlimb withdrawal threshold in OA and RA models.^23^, ^86^, ^127^, ^144^ TRPV1 agonists, such as capsaicin and RTX, can reduce pain in arthritis after pre‐treatment,^103^, ^126^, ^149^, ^150^ which is associated with the specific desensitization,^151^ defunctionalization^152^ and structural ablation^153^ of nerve endings (Table 2). Long‐term use or high doses of the agonists capsaicin or RTX can act on the terminals of nociceptive fibres expressing TRPV1, activate TRPV1 and mediate a large influx of Ca^2+^. This leads to long‐term defunctionalization of its nerve terminals without responding to further deleterious stimuli and resulting in long‐lasting analgesic effects.^149^, ^164^ However, the role of TRPV1 in arthritis pain is also controversial. Some studies have reported that systemic administration of the TRPV1 inhibitor AZD1386 has no effect on pain management in individuals with knee OA compared to administering the placebo.^165^ In contrast, another study reported that AZD1386 failed to relieve pain in a rat model of OA.^166^ Furthermore, Okun et al. found that the TRPV1 antagonist AMG981 did not affect the persistent pain caused by advanced MIA.^145^ It is possible that TRPV1 expression in the DRG is increased mainly in the early stages of MIA‐induced OA, whereas TRPV1 has no effect on mechanical nociception or does not play a dominant role in persistent pain at later time points.^167^ The discrepancies in these studies may reflect the drug effects on different animal models, on different types of pain, such as acute and chronic pain or on different stages of pain, such as early and late stages of pain.

**TABLE 1:** Effects of TRPV1 channel drugs in preclinical models.

Type of models	Exp. animals	Drugs	Treatments	Effects	Refs.
Carrageenan‐induced knee‐joint RA	Male Sprague–Dawley rats	RTX	30 μL (0.0003% or 0.003%) for 8 days; single intra‐articular administration.	Pain score↓; Limp and weight distribution ratio↓; Joint swelling↓	142
MIA‐induced OA	Male Sprague–Dawley rats	A784168; A795614	A784168 (3, 10 or 30 μmol/kg) or A795614 (30, 100 or 300 μmol/kg); oral administration; A784168 or A795614 (10, 30,1 00 nmol); intrathecal administration.	Weight‐bearing differences↓; mechanical allodynia↓; thermal hyperalgesia↓	114
MIA‐induced OA	Male Sprague–Dawley rats	ABT‐102; A993610	ABT‐102 (3 μmol/kg or 10 μmol/kg) or A993610 (12 μmol/kg) twice daily for 12 days; oral administration.	Inflammatory pain↓; weight‐bearing differences↓; grip force↑	143
MIA‐induced OA	Sprague–Dawley rats	Capsaicin	Pretreatment with 100 μL 0.5% capsaicin for 14 days; intra‐articular administration.	Mechanical hyperalgesia↓; weight‐bearing defect of the hind paw↓; paw ambulation scores↑; subchondral bone erosions and destruction in knee joints↓	103
MIA‐induced OA	Male Sprague–Dawley rats	A995662	Single administration of 3, 10, 30 or 100 μmol/kg; oral administration; Repeated administration of 15 μmol/kg (twice per day) for 12 days; oral administration.	Pain↓; hind limb grip force↑; basal spinal release of CGRP and glutamate ↓	127
MIA‐induced OA	Male Sprague–Dawley rats	A889425	10, 30, 100 or 300 μmol/kg; oral administration; 10 and 30 μmol/kg, intravenous administration.	Behavioural grip force↑; the evoked firing of nociceptive specific neurons in OA↓	144
MIA‐induced OA	Male Sprague–Dawley rats	AMG9810	30 mg/kg, intraperitoneal administration.	Thermal hyperalgesia↓; the weight‐bearing shift induced by a 3 mg dose of MIA↓	145
MIA‐induced OA	Male Sprague–Dawley rats	JNJ‐17203212	1 mg/50 μL; intra‐articular injection; 1 mg/300 μL, i.p. injection; 0.075 or 0.15 mg/100 μL; intra‐arterial injection; 18.75, 37.5 or 75 μg/50 μL; topical spinal injection.	Weight‐bearing asymmetry↓; mechanically evoked responses of knee joint afferents and dorsal horn wide dynamic range neurons ↓	23
MIA‐induced OA	Male Sprague–Dawley rats	RTX	50 μL (0.0003%, 0.003% or 0.03%) for 21 days; intra‐articular injection.	Weight‐bearing↑; mechanical allodynia↓; thermal hyperalgesia↓; expression of CGRP in DRG↓	126
Carrageenan‐induced RA	Male C57BL/6 mice	CapsaicinRTX	Pretreatment with 0.01% Capsaicin, 0.001% RTX or 0.0003% RTX for 7 days; intra‐articular injection.	Evoked pain scores↓; antinociception↑; dynamic weight bearing↑	146
OA	Dogs	RTX	Single intra‐articular injection of 10 μg/100 μL.	Pain scores↓; gait and weight‐bearing↑	147
Collagen‐antibody‐induced RA	Male C57BL/6 mice	RTX	Pretreatment of 30, 70 or 100 μg/kg for 3 consecutive days; subcutaneous injection.	Mechanical hyperalgesia↓; cold allodynia↑; joint oedema and arthritis severity↓; bone density and trabecular bone thickness↑	104
CFA‐induced RA; MIA‐induced OA	Male Sprague–Dawley rats	APHC	0.01, 0.05, 0.1 or 1 mg/kg; subcutaneous administration.	Joint swelling↓; pain‐induced behaviour↓; articular cartilage destruction↓; IL‐1β concentration in synovial fluid↓	86
OA	Male and female C57BL/6 mice	Capsaicin	0.5 and 1.0 mg/kg five times per week for 12 weeks; i.p. injection.	DMM‐induced pain in male mice↓; TRPV1 expression in DRG of male mice↓; joint cartilage degeneration has not changed	148

Abbreviations: ↑, increased; ↓, decreased; CFA, Complete Freund's adjuvant; CGRP, calcitonin gene‐related peptide; DMM, destabilized medial meniscus; DRG, dorsal root ganglion; IL‐1β, interleukin‐1β; MIA, monosodium iodoacetate; OA, osteoarthritis; RA, rheumatoid arthritis; RTX, resiniferatoxin.

**TABLE 2:** The Functional mechanism of TRPV1 channel drugs in different treatment patterns or drug concentrations.

Drugs	Treatment patterns/drug concentrations	Functional mechanism	Refs.
TRPV1 agonists	A single intrathecal injection	Depletion of substance P from primary sensory neurons.	16, 154, 155, 156
	Concentration of treatment	TRPV1 activation induces Ca^2+^ influx and inactivation of voltage‐dependent sodium channels, voltage‐dependent calcium channels and Piezo2. Inactivation of these channels causes a decrease in their ability to generate and propagate action potentials and leads to transient analgesia.	157, 158
		TRPV1 activation induces Ca^2+^ influx, leading to receptor desensitization of TRPV1 ion channels. Desensitization of the TRPV1 receptor reduces the response of TRPV1 to endogenous ligands or inflammatory mediators, which in turn reduces TRPV1‐related hyperalgesia.	
	Single high‐concentration or multiple low‐concentration	TRPV1 activation and influx of large amounts of Ca^2+^, leading to the activation of Ca^2+^‐dependent protease calpain. Ablation of nerve terminals by calpain activation results in long‐term defunctionalization of TRPV1 expressing afferents and mediates long‐lasting analgesia for chronic pain.	157, 159, 160
TRPV1 antagonists	Concentration of treatment	Blocking channel pores and inhibiting heat‐ and proton‐induced Ca^2+^ influx in sensory neurons, thereby reducing inward current generation or neuropeptide release. In addition to TRPV1, capsazepine also blocks voltage‐sensitive calcium channels and nicotinic acetylcholine receptors, ultimately exerting analgesic effects.	23, 161, 162, 163

Systemic administration of TRPV1 antagonists induces hyperthermia in both humans and animals.^168^, ^169^ Under normal conditions, neurons expressing TRPV1 in internal organs become overactivated to inhibit skin vasoconstriction. However, systemic administration of TRPV1 antagonists leads to the desensitization of these neurons resulting in cutaneous vasoconstriction and subsequent hyperthermia.^170^, ^171^ Notably, TRPV1 KO mice did not exhibit severe dysregulation in thermo‐regulation,^172^ suggesting that other mechanisms also play a role in maintaining a relatively stable body temperature. Furthermore, in an OA pain model, local administration of TRPV1 antagonist JNJ‐17203212 produced the same analgesic effect as systemic administration and did not affect core body temperature.^23^ Concerning the route of administration, topical capsaicin creams or high‐dose capsaicin‐containing patches have been approved as effective adjuvant therapy for the insistent pain of post‐herpetic neuralgia and in the treatment of OA, but their use has been limited by the relatively weak efficacy of capsaicin creams, the need for multiple applications and skin irritation.^173^ Topical capsaicin creams can be used in combination with other analgesics, providing a low‐risk choice for patients who cannot achieve pain control using other regimens. Oral or intraperitoneal administration has been widely introduced in preclinical trials but is not an ideal option, given the adverse effects of capsaicin on blood pressure, respiration and other reflex pathways and the potential for increased body temperature and cardiovascular complications associated with TRPV1 antagonists.^174^, ^175^ In addition, TRPV1 antagonists can exert potent analgesic effects by blocking the centrally located TRPV1 receptors. Some drugs (e.g., A‐425619 and A‐795614) have low central nervous system penetration, so intrathecal analgesia is better than oral administration.^103^, ^114^ The intrathecal injection can achieve the same analgesic effect as an intra‐articular injection, and intrathecal injection can also alleviate the pain caused by local inflammatory factors within the joint and surrounding soft tissues.^176^ Intra‐articular therapeutic strategies appear well suited for the management of OA, in particular, the knee OA. Intra‐articular RTX suppressed the carrageenan‐induced oedema by at least one‐third, and its smaller therapeutic dose was approximately 1000 times lower than the dose producing analgesia following systemic administration of RTX in rats.^142^, ^177^ Additionally, intra‐articular administration of an analgesic dose of TRPV1 agonist or antagonist could reduce many adverse effects, such as hyperthermia and the unnecessary loss of C‐fibre in the non‐target tissues.

There is growing evidence of sex differences in TRPV1 sensitivity. Clinical studies have shown gender disparities in TRPV1‐mediated pain responses in various body regions, including the back of the hand, ankle, dental pulp and forehead.^178^, ^179^, ^180^ The results of Bai et al. suggest that testosterone may regulate TRPV1 expression and affect pain responses in the CFA‐induced chronic pain model.^181^ In another study related to OA, a high‐dose TRPV1 antagonist, capsazepine (30 mg/kg), was found to reverse mechanical pain abnormalities more significantly in male OA mice than in female mice. Additionally, down‐regulation of TRPV1 expression was observed in the DRG tissues of male after treatment, but not in female mice. These findings indicate the presence of sex differences in the regulation of TRPV1 expression by capsazepine.^148^ Most current studies on the role and mechanisms of TRPV1 channels in arthritis pain have primarily involved male rodents. Therefore, it is essential to identify sex‐specific genes related to TRPV1 and elucidate the underlying mechanisms of gender differences in the treatment of arthritis pain.

## 7 CLINICAL APPLICATION OF TRPV1‐TARGETING DRUGS IN ARTHRITIS

TRPV1 agonists and antagonists are currently used in the treatment of arthritis pain. TRPV1 antagonists act directly on TRPV1 receptors to reduce pain‐related signal transduction and neuropeptide release, thereby inhibiting the generation of pain. The analgesic effect of TRPV1 agonists (such as capsaicin) can be maintained for weeks or even months through desensitization, neuropeptide depletion and nociceptor terminal ablation. However, the pain is restored due to the regeneration of nociceptor nerves after the application of capsaicin.^182^ Since TRPV1 and TRPA1 are co‐expressed in neurons, capsaicin‐induced ablation of nociceptor terminals may affect TRPA1‐mediated pain and inflammation.^183^, ^184^ Therefore, capsaicin treatment can block all functions of TRPV1 expressing nociceptors, providing individuals with a more effective therapeutic effect. Here, we summarize the current TRPV1 channel drugs mainly used for arthritis treatment (Table 3).

**TABLE 3:** TRPV1 channel drugs for the treatment of arthritis pain.

Type of arthritis	Number of patients	Drugs	Treatments	Effects	Refs.
RA, OA	70 (OA patients) and 31 (RA) patients	Capsaicin cream	0.025% capsaicin cream or placebo four times daily; 4 weeks	RA and OA patients demonstrated 57% and 33% reductions in pain, respectively.	29
OA	167	Capsaicin glyceryl trinitrate	placebo, 0.025% capsaicin, 1.33% glyceryl trinitrate or 0.025% capsaicin and 1.33% glyceryl trinitrate four times daily; 6 weeks	Glyceryl trinitrate enhanced the analgesic effect of capsaicin; A combination of capsaicin and glyceryl trinitrate reduced the burning sensation.	185
OA	611	Civamide cream	civamide cream 0.075% group applied on average 2.4 g/day and civamide cream 0.01% (control) group applied on average 2.6 g/day, three times daily; 12 weeks and extension	The treatment effect of the civamide cream 0.075% group was better than that of the civamide cream 0.01% (control) group.	186
OA	191	AZD1386	30 mg or 90 mg AZD1386 or placebo; 4 weeks	Elevated alanine aminotransferase and aspartate aminotransferase; No treatment differences in WOMAC pain	165
OA	33	Mavatrep (JNJ39439335)	A single dose of mavatrep (50 mg), naproxen (500 mg) or placebo was administered twice daily	Pain, stiffness and physical function were significantly reduced.	187
OA	24	Mavatrep (JNJ39439335)	10, 25 or 50 mg mavatrep or placebo, once a day	The pain was significantly reduced after stair climbing.	188
OA	54	NEO6860	NEO6860 (500 mg), naproxen (500 mg) or placebo twice daily; 1 day	The NEO6860 treatment group had a trend of analgesia, but the difference was not statistically significant compared with the placebo group.	189
OA	172	CNTX‐4975	Single intra‐articular injection of a placebo, CNTX −4975 0.5 mg or CNTX‐4975 1.0 mg; 24 weeks	OA–associated pain decreased at week 12 in the 0.5‐mg group and at week 24 in the 1.0‐mg group.	190

Abbreviations: OA, osteoarthritis; RA, rheumatoid arthritis; WOMAC, Western Ontario and McMaster Universities Arthritis Index.

## 8 TRPV1 AGONISTS

In a 4‐week, double‐blind, randomized, multi‐centre study, the use of topical capsaicin cream showed a reduction in pain among patients with OA when compared to a placebo. However, it did not provide pain relief for individuals with RA. It should be noted that the treatment site experienced a transient burning sensation as a side effect of the capsaicin cream.^29^, ^191^ The analgesic effect of combined topical capsaicin and nitroglycerin was increased compared with capsaicin alone.^185^ There was also evidence that topical capsaicin was superior to placebo in terms of pain relief and that topical NSAIDs and topical capsaicin provided comparable pain relief efficacy.^192^ Civamide is another TRPV1 receptor agonist with a mechanism of action similar to capsaicin. The continuous application of 0.075% civamide showed positive effects on pain reduction and Subject Global Evaluation (SGE) in individuals with OA for a duration of 1 year. Moreover, the use of civamide cream does not result in systemic absorption and is considered safe based on its profile.^186^

CNTX‐4975 is a highly purified trans‐capsaicin. The efficacy and safety of a single intra‐articular injection of different doses (0.5 mg or 1.0 mg) of CNTX‐4975 were recently evaluated in a randomized, double‐blind, placebo‐controlled, dose‐ranging, Phase IIb study involving 175 patients with knee OA who were clinically assessed over a period of 24 weeks.^190^ Significant improvements were observed in individuals treated with 1 mg of CNTX‐4975 across various parameters, including the Western Ontario and McMaster Universities (WOMAC) Osteoarthritis Index, pain while walking scores, joint stiffness and joint function when compared to the placebo group. These positive treatment effects were sustained for up to 24 weeks. This study found no significant difference in the incidence of treatment‐emergent adverse events between the placebo and CNTX‐4975 groups, indicating that the use of CNTX‐4975 does not result in serious safety concerns.^190^

## 9 TRPV1 ANTAGONISTS

JNJ‐39439335 also known as mavatrep, functions as an antagonist for the TRPV1 receptor. A Phase 1b study was conducted in which individuals with OA were administered a single dose of 50 mg mavatrep, 500 mg naproxen twice daily or a placebo. The results demonstrated that the individuals treated with mavatrep showed significant pain reduction, as measured by pain after climbing stairs (PASC), Numerical Rating Scale (NRS) score and WOMAC score. However, some adverse events emerged in the mavatrep group, including cold sensation, decreased heat sensation, dysgeusia, paresthesia and heat sensation.^187^ Another subsequent 10‐week clinical study observed that repeated administration of mavatrep did not lead to tolerance.^188^

Another TRPV1 receptor antagonist, NEO6860, was studied in a Phase II trial involving individuals with OA. The individuals received a one‐day treatment (two doses) of NEO6860 (500 mg twice daily), naproxen (500 mg twice daily) or a placebo. The assessment of knee pain intensity revealed that individuals treated with NEO6860 and naproxen experienced less pain at 3 and 24 h after the staircase pain test. However, statistically significant differences between NEO6860 and placebo treatment were only observed at the 24 h time point. Adverse events were also reported in the NEO6860 treatment group, including headache, nausea, dizziness, fatigue, increased blood pressure/hypertension and hypoesthesia, which raised safety concerns.^189^

While several TRPV1 agonists or antagonists have demonstrated improved efficacy in pre‐clinical and clinical trials for arthritis pain, attention needs to be given to adverse reactions, such as drug‐induced increases in core body temperature. TRPV1 channels in nociceptive sensory neurons can undergo conformational changes triggered by proton, thermal and chemical ligands. TRPV1 antagonists that have a minimal impact on proton‐induced gating can effectively avoid the adverse effects of hyperthermia.^193^, ^194^ Consequently, the multi‐steric gating feature of TRPV1 channels presents a new approach for developing potent TRPV1 antagonists without the risk of hyperthermia.^141^ Additionally, exploring other therapeutic strategies, such as targeting both TRPA1 and TRPV1 or the fatty acid amide hydrolase and TRPV1, may yield more effective analgesic effects compared to TRPV1 antagonists alone.^167^, ^195^

## 10 CONCLUSION AND FUTURE DIRECTIONS

**FIGURE 2:**
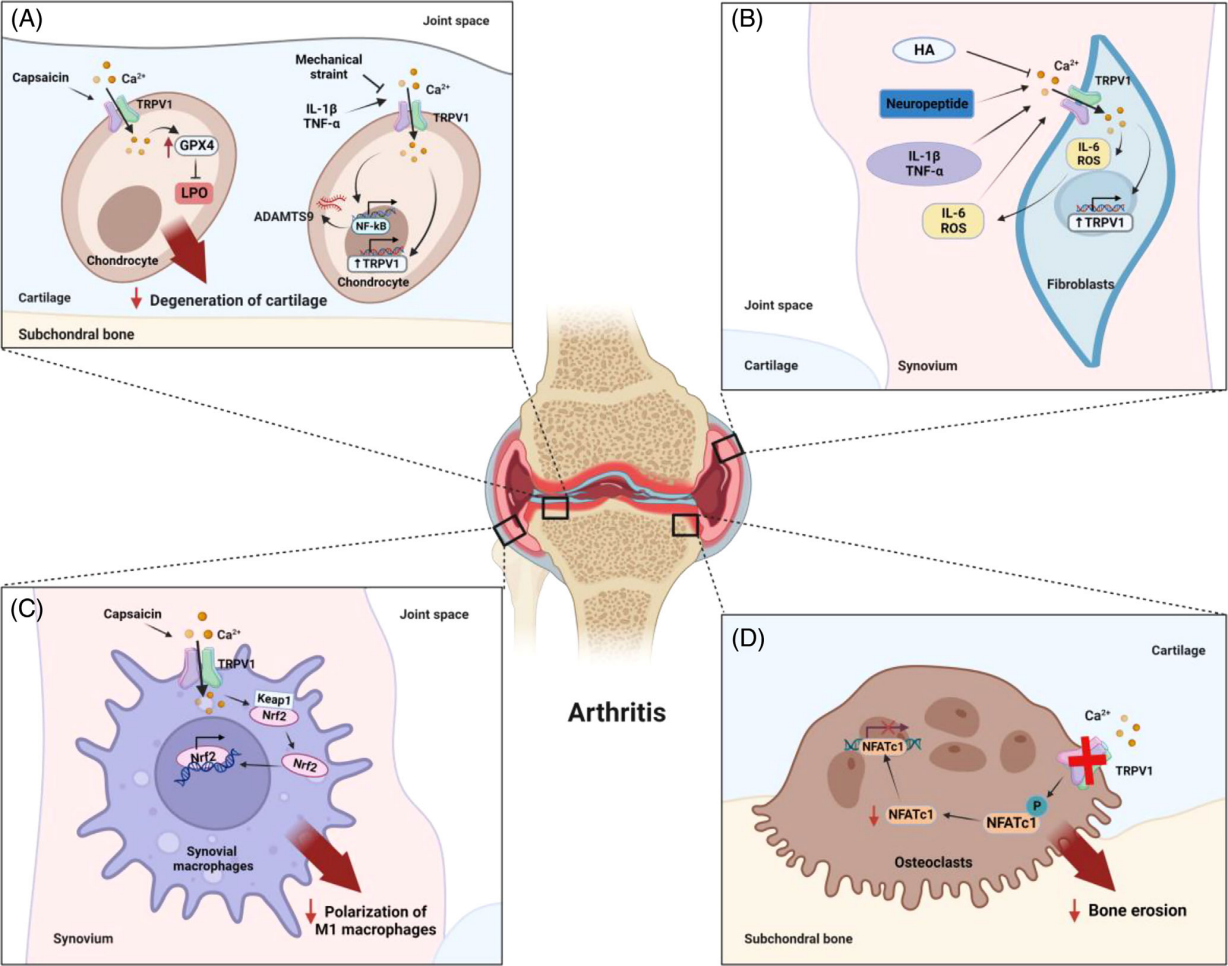
The role of TRPV1 on different cell types of the joint during the onset and progression of OA. (A) The anti‐ferroptosis effect of capsaicin through the TRPV1‐Glutathione peroxidase 4 (GPX4) pathway in OA chondrocytes. Mechanical stress reduces intracellular Ca^2+^ concentration through TRPV1 channels leading to a cytokine‐induced decrease in A Disintegrin and Metalloproteinase with Thrombospondin Motif (ADAMTS) 9 expression. In addition, the activation of TRPV1 on chondrocytes can potentially induce an upregulation of TRPV1 gene expression. Similarly, the activation of TRPV1 on synovial cells can trigger the release of inflammatory factors, the production of reactive oxygen species (ROS) and an increase in TRPV1 gene expression. Conversely, inflammatory factors, ROS and neuropeptides promote TRPV1 activation. (B) Hyaluronic acid (HA) in synovial fluid inhibits the opening of TRPV1 channels and reduces Ca^2+^ influx into cells. (C) Capsaicin inhibits the polarization of M1 macrophages through TRPV1 channels, reduces the release of pro‐inflammatory factors and thus alleviates the progression of OA. (D) Inactivation of TRPV1 channels inhibits Ca^2+^ influx and the expression of its downstream transcription factor nuclear factor of activated T cells c1 (NFATc1), leading to reduced osteoclastogenesis, reducing bone loss in early OA.

The TRPV1 channels play a crucial role in nociception during arthritis, and numerous studies have observed an upregulation of this channel on sensory nerves in relation to arthritis pain. TRPV1 channels are widely expressed in histiocytes, nerves and brain regions that are relevant to arthritis pathophysiology. They are involved in regulating chondrocyte ferroptosis, synovial inflammatory response, macrophage polarization and bone homeostasis (Figure 2). Additionally, TRPV1 channels play a vital role in nerve sensitization, defunctionalization or central sensitization, and they can release various neuropeptides that contribute to arthritis development. Both pre‐clinical and clinical data have indicated that TRPV1 agonists, when administered as a pre‐treatment, may have the potential to prevent or slow down the progression of OA. Consequently, targeting the TRPV1 channels holds significant promise as a therapeutic approach for arthritis treatment and the management of arthritic pain.

**FIGURE 3:**
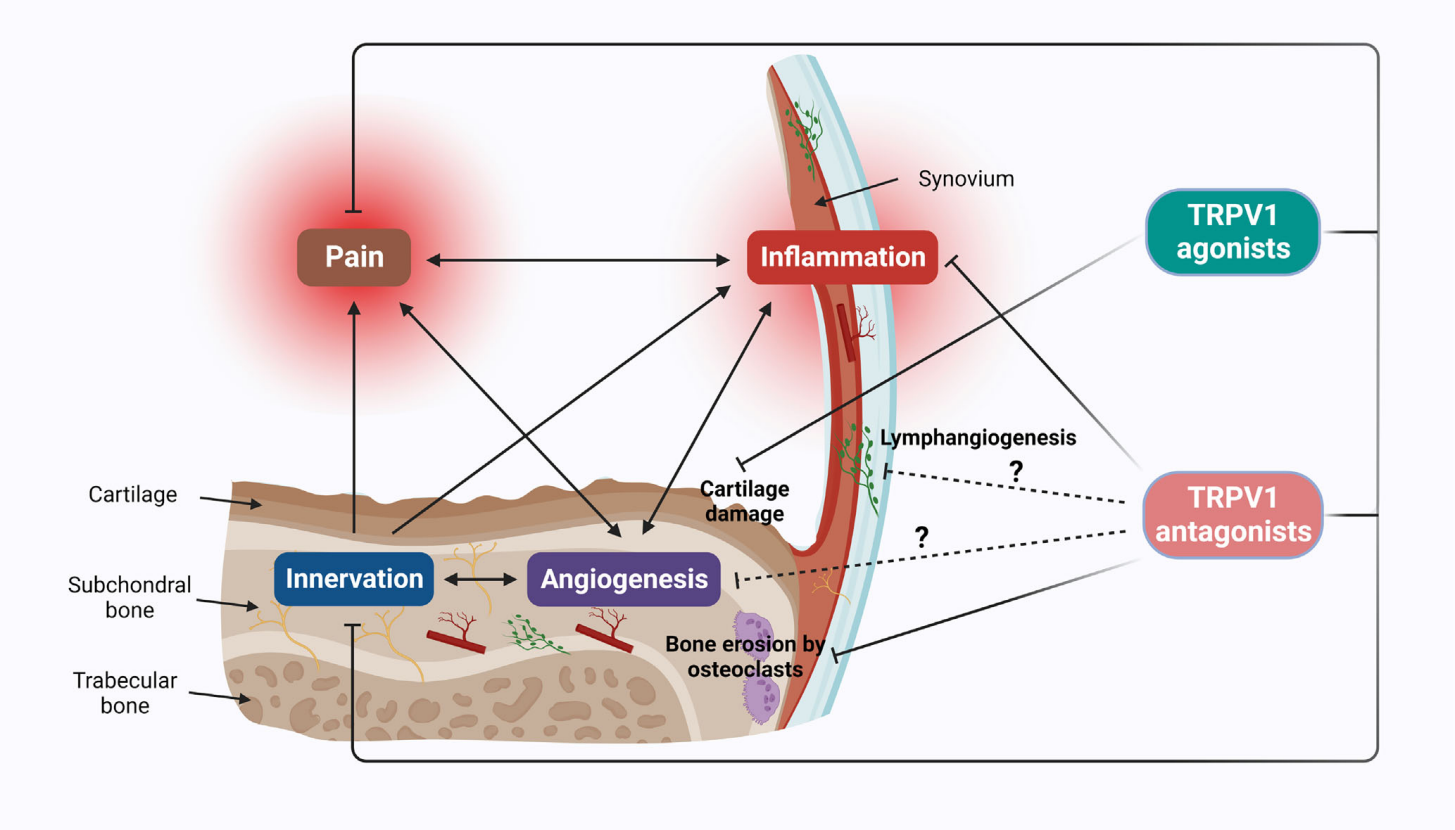
The relationship between angiogenesis, lymphangiogenesis, innervation, pain and joint destruction. The schematic diagram of the hypothetical molecular mechanism of the therapeutic potential of TRPV1 agonists and antagonists in the treatment of OA and RA.

Although great progress has been made in the research of TRPV1 channels in OA and RA, there are still some limitations. In order to make better use of TRPV1 channels for the treatment of arthritis, the following problems need to be addressed. First, the contribution of TRPV1 to angiogenesis and lymphangiogenesis in arthritis pathogenesis should be assessed. Synovial angiogenesis in RA and OA promotes the invasion of inflammatory cells and increases the distribution of joint pain receptors, accelerating the degeneration of articular cartilage and the occurrence of pain.^76^ Some clinical drugs, such as simvastatin and erythropoietin, induce endothelial nitric oxide synthase phosphorylation and increase NO synthesis in endothelial cells by activating TRPV1‐mediated Ca^2+^ influx, ultimately promoting angiogenesis.^196^, ^197^ Lymphatic endothelial cells (LECs) express various acid‐sensing receptors, acidic conditions (pH = 6.4) induce IL‐8 expression through activation of TRPV1 in LECs, treatment with the TRPV1 antagonist I‐RTX inhibited the expression of IL‐8.^198^ Serum IL‐8 plays a role in knee OA, and its expression levels are positively correlated with increased knee symptoms, infrapatellar fat pad signal intensity alteration, and serum levels of bone or cartilage biomarkers.^199^ In addition, it has been reported that capsazepine could prevent the hypertonic environment‐induced decrease of contraction rate and lymph flow by blocking the TRPV1 channel, indicating that TRPV1 is particularly important in regulating the rate of spontaneous contraction of lymphatic vessels and lymph flow.^200^ It is possible to alleviate arthritis pain by inhibiting angiogenesis and lymphangiogenesis through the inhibition of TRPV1 activity (Figure 3).

Consequently, unravelling the link between TRPV1 and angiogenesis or lymphangiogenesis in arthritis holds significant implications for comprehending the pharmacological mechanisms of TRPV1 and identifying novel therapeutic targets for arthritis. Additionally, TRPV1 plays multiple roles in arthritis. Activation of TRPV1 in cartilage protects chondrocytes from degeneration, while inhibition of TRPV1 in synovial tissue of joints inhibits synovial inflammatory responses. Therefore, targeted TRPV1 therapy should be carefully and pragmatically considered as the balance between the beneficial and the adverse effects, and the choice of treatment should be tailored to each patient's specific condition, disease severity and treatment goals. Selectively targeting specific tissue or spinal cord nerve endings using drugs may avoid some systemic adverse effects, such as targeting the expression of the TRPV1 channel using genome‐editing tools, which could block specific cell or certain populations of TRPV1^+^ neurons and thus be much better tolerated. MiR‐375 and miR‐455 were identified to repress TRPV1 expression via targeting the 3’‐UTR of TRPV1 mRNA, which has been shown to be downregulated in patients with low back pain with neuropathic pain,^201^ so they may also function as therapeutic targets to indirectly modulate TRPV1 activity in arthritis. Furthermore, employing TRPV1‐RNAi as a potential therapeutic approach could effectively target the TRPV1 receptor to address pathological alterations in arthritis and preserve essential physiological defence mechanisms. Small interfering RNA (siRNA) therapy can not only target mRNA to silence the expression of catabolic genes to suppress the inflammatory response of arthritis but also silence the expression of genes that inhibit tissue regeneration by improving cartilage and bone tissue regeneration.^202^ Several studies have shown that siRNA targeting TRPV1 can be used to treat dry eye disease, hyperalgesia induced by Complete Freund's Adjuvant and bone cancer pain.^203^, ^204^, ^205^ Targeting TRPV1 using TRPV1 siRNA to reduce protein expression by inhibiting mRNA expression has become a method for evaluating gene‐specific activity.^206^ Ultimately, the involvement of TRPV1 in arthritic conditions remains a subject of debate. It is crucial to exercise caution in the study design and result analysis when investigating TRPV1 channels, particularly in terms of agonist or antagonist dosage, treatment duration, TRPV1 expression distribution, potential splicing variants and functional implications. Taking these factors into account will contribute to a more comprehensive understanding of the role of TRPV1 in arthritic diseases.

In summary, the involvement of TRPV1 in the development of arthritis highlights its significance in the disease's pathogenesis. Further research and drug development efforts are essential to ensure the safety and effectiveness of TRPV1 as a therapeutic target. Anticipated future studies will shed more light on the precise role and regulatory mechanisms of TRPV1 in arthritis. This knowledge will establish a solid foundation for advancing targeted TRPV1 therapies and their clinical applications.

## AUTHOR CONTRIBUTIONS

Di Chen, Liping Tong and Yan Chen contributed conception and design of the study; Zhidong Liao wrote the first draft of the manuscript and prepared the figures and tables; Muhammad Umar and Xingyun Huang wrote sections of the manuscript; Ling Qin, Guozhi Xiao, Liping Tong and Di Chen provided the critical revisions. All authors read and approved the final manuscript.

## FUNDING INFORMATION

This work was supported by the National Key Research and Development Program of China (2021YFB3800800) to Liping Tong and Di Chen. This project was also supported by the National Natural Science Foundation of China (NSFC) grants 82030067 and 82250710174 to Di Chen. This project was also supported by the NSFC grant 82161160342 to Di Chen and Ling Qin.

## CONFLICT OF INTEREST STATEMENT

The authors declare no conflict of interest.
